# A web-based geographic information system monitoring wildlife diseases in Abruzzo and Molise regions, Southern Italy

**DOI:** 10.1186/s12917-023-03727-9

**Published:** 2023-10-02

**Authors:** Alessio Di Lorenzo, Valentina Zenobio, Daniela Cioci, Francesca Dall’Acqua, Susanna Tora, Simona Iannetti, Marco Rulli, Daria Di Sabatino

**Affiliations:** Istituto Zooprofilattico dell’Abruzzo e del Molise “G. Caporale”, Teramo, Italy

**Keywords:** Diseases control, Wildlife, Geographic Information Systems, Web-GIS, Italy

## Abstract

**Background:**

Nowadays there is a worldwide consensus on the importance of conducting wildlife disease surveillance. Indeed, 60% of emerging infectious diseases are zoonotic in nature, and the majority of these (71.8%) originate in wildlife. Surveillance of wildlife diseases is crucial to prevent negative effects on human and animal health. Data digitization and sharing are among the main goals for the present and coming years. Geographic Information Systems (GIS) are increasingly used to analyze the geographical distribution of diseases and the relationships between pathogenic factors and their geographic environments.

**Methods:**

Wild animal’s samples collected in the Abruzzo and Molise regions and delivered to our laboratory are entered in our Laboratory Information System and processed to be displayed through the Web-GIS mash-up presented in this paper. We built it using both open source and proprietary solutions, to produce data driven interactive maps, charts and tables to help to understand the epidemiology of wild animal diseases, their spread and trend.

**Results:**

Since 2013, 9.606 samples collected from wild animals have been analyzed in the laboratories of the IZS-Teramo and have been recorded in the system, facilitating the reporting to the judicial authorities and the identification of highly risky areas to set up control and repression measures. Moreover, thanks to the monitoring health protocol, a canine distemper epidemic in wolves has been detected and monitored in its temporal and spatial evolution, as well as cases of bovine tuberculosis in wild boars.

**Conclusions:**

While it is more evident that the starting point is to choose the right sampling method, it is for sure less obvious that the information system in which data is stored is equally important. In fact, it should give the possibility to consult it in an easy and instructive way. GIS allows immediately grasping the spatial relationships between the data itself and those between the data and the territory; it is an important tool to support veterinary services in managing epidemic and non-epidemic emergencies and performing epidemiological investigations, but also to examine control plans and identify new gaps and challenges.

**Supplementary Information:**

The online version contains supplementary material available at 10.1186/s12917-023-03727-9.

## Background

Nowadays there is a worldwide consensus on the importance of conducting wildlife disease surveillance. Indeed, 60% of emerging infectious diseases are zoonotic in nature, and the majority of these (71.8%) originate in wildlife [1]. Coronaviruses, HIV, Ebola, H5N1, H1N1 are the more recent zoonoses emergencies that have in common a wild animal origin [1]. There is a growing understanding of the necessity of disease surveillance in free-ranging wildlife due to the growing awareness of the effects of developing diseases on both humans and animals as well as the significance of wild animals as hosts and/or reservoirs of zoonotic pathogens [2]. The first indication of a new health threat may come from reports of unexpected diseases or deaths in wildlife populations. For instance, passive surveillance on wild bird mortality acts as a sensitive indicator for the circulation of West Nile Virus and the risk of infection for humans [3].

Diseases can be driven in wild populations in different ways such as by the exposure to an infectious agent from another wild population as well as a domestic one, or by changes in the intrinsic structure of the population itself which are capable to increase its vulnerability (i.e. overcrowding and stress due to habitat modification, dietary shifts, genetic instability, climate changes) [4, 5]. Although it is well recognized that infections in wild animals can pose a risk to human health, domestic animals and endangered species causing loss in terms of economy and human lives, the surveillance of agents causing emerging diseases in wild animals is rarely a national priority.

It is important to remember that disease surveillance/monitoring consists of two components: the “numerator data,“ i.e. the number of diseased people, and the “denominator data,“ i.e. the size of the target population. While active surveillance/monitoring is necessary to evaluate epidemiological dynamics and the success of control measures on endemic diseases, passive disease surveillance/monitoring increases the possibility of early detection of new diseases. In order to monitor pathogen and population trends in wildlife, it is necessary to build integrated and coordinated techniques [6]. Collecting data on wild animals, both on health status and population structure, is expensive and time consuming, especially in the case of effective systems. Moreover, due to the reporting-bias, frequently unquantified, and the lack of knowledge on “denominator data ‘’, the difficulty in interpreting surveillance results has improved [7]. Passive surveillance, for its characteristics, is heavily affected by all the limitations described above, nonetheless, it remains the major source of health data in wildlife worldwide. The reason is that passive surveillance is the best choice to determine which diseases are present in the wild population and to early detect new pathogens if the diseases cause evident clinical symptoms [8].

Data digitization and sharing are among the main goals for the present and coming years. In this framework, disease information systems are able to generate and process information useful for timely and efficient delivery of alerts helpful for control activities as well as for monitoring disease trends. At the international level, since 2005, the development of the World Animal Health Information System (WAHIS) has allowed access to the notification of data on wildlife disease events dutifully reported to the World Organisation for Animal Health (WOAH) by the member countries. Since 2008, a subsection named WAHIS-Wild, has been launched. WAHIS-Wild is exclusively dedicated to diseases and it is useful for monitoring disease threats to wildlife (including those of potential public health and conservation concern) without impacting the international trade of animals or animal products. However, WAHIS and WAHIS-Wild, and other public international databases, collect and share data only on confirmed disease events, and do not consider data on surveillance activities.

Geographic Information Systems (GIS) and related technologies, like remote sensing, are increasingly used to analyze the geographical distribution of diseases, as well as relationships between pathogenic factors (causative agents, vectors, and hosts) and their geographic environments [9]. GIS are able to integrate and analyze spatially referenced data, and through the investigation of spatial patterns of occurrence improve the interpretation of wildlife disease surveillance outcomes [10]. Epidemiologists use GIS to assess proximity, aggregation and clustering, as well as to perform spatial smoothing, interpolation and spatial regression [11]. GIS has also been used extensively in epidemiology for disease surveillance and intervention monitoring [12]. By mapping disease cases in geographic space, local and national governments can easily identify the distribution and spread of disease across geographic regions, optimize the planning of intervention locations, and monitor their effectiveness [11].

GIS enables remixing and repurposing of data by “mashing up” various data and map layers or themes from multiple sources into a single map (with multiple layers covering the same locations superimposed like an onion’s skin). With the advent of Web 2.0 technologies, the democratization and participatory nature of GIS have never been more possible or powerful [13].

In this paper we describe the Web-GIS application we developed to monitor the epidemiology of wild animal diseases, their spread and trend in Abruzzo and Molise, two regions located in southern Italy. This activity has been carried out in the framework of the regional surveillance programmes of Abruzzo and Molise to collect, retrieve, share and display epidemiological data and spatial distribution of wildlife surveillance outcomes for local veterinary services.

## Methods

Samples collected in Abruzzo and Molise regions from wild animals are delivered to the Sample Acceptance and Control Unit of the Istituto Zooprofilattico Sperimentale dell’Abruzzo e del Molise “G. Caporale” of Teramo (IZS-Teramo), together with a specific and standardized document, named Laboratory Requisition Form (LRF), filled in by the veterinarians or by the Competent Authority (LFR is provided in the additional file n°1). The LRF contains information on the wild animal sampled (species, sex, age), site of collection (province, village and coordinates), the suspected cause of death, and the sampling context (i.e. investigation on the cause of death, health monitoring of hunted animals, translocation, study). All the data reported in the sampling LRF are entered by the Sample Acceptance and Control Unit’s operators in a specific and dedicated section of the Laboratory Information Management System of the IZS-Teramo (SILAB). This section, named “Information system for wild animals”, was implemented in 2013, together with a dedicated operational workflow, with the aim of collecting information on the local wildlife disease status, by storing it into an Oracle Database Management System (DBMS).

We also developed a monitoring health protocol addressing the diagnostic test to be carried out on the sample in relation to the species of origin and to the material committed to the Sample Acceptance and Control Unit (for the detailed health monitoring protocol see the additional file n° 2).

**Fig. 1:**
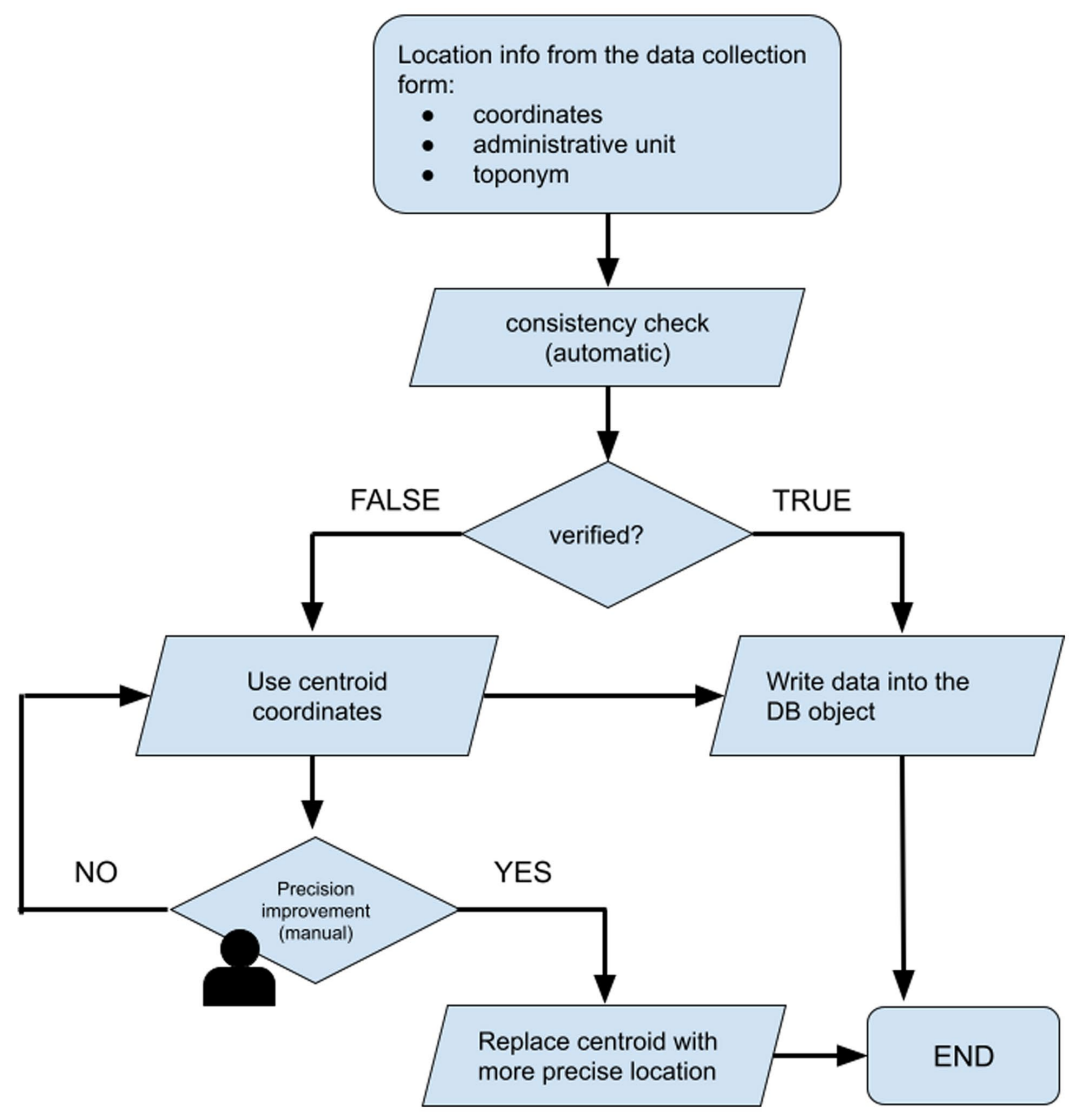
Diagram of geographic data acquisition and correction workflow

Data entered in SILAB are routinely checked by epidemiologists for possible errors and missing information. A daily based control is performed on the completeness and quality of data collected. With regard to the geographic coordinates, an automatic procedure, triggered by the data entry, checks against an administrative units layer of reference, based on an official ISTAT (National Institute of Statistics) dataset [14], if the coordinates of a sampling fall inside the administrative unit (AU) of finding (consistency check). In case of inconsistency, the provided coordinates are automatically replaced by those of the centroid of the AU. Data assigned to the centroid are checked daily by epidemiologists that, using additional information as toponym registered in LRF or provided by the surveyor (client in the LRF), try to retrieve the precise location, improving data quality. In those cases in which coordinates cannot be made more precise, the centroid has been used as an approximation of the real data (Fig. 1).

We used two different database objects to store, respectively, sampling and result data which is then displayed in the Web-GIS. The first one contains all data collected in SILAB through the LFR (infromarion characterizing the sample or sampled animal and useful for epideiological analysis, such as “wild/captive, finding cirucmstances, suspect cause of death, place of finding, clinical signs presence, sampling reason, animal gender and age”). The second database object contains, for each sample, the analytical method, the laboratory test performed, and the outcome of the diagnostic test.

We developed the Web-GIS application using both open source and proprietary solutions. It is a mashup [15], namely a web application that combines data from multiple sources to form a new integrated resource. In this case, data provided by a typical client-server architecture are spatially overlapped to general purpose spatial data provided by the Esri Inc. cloud service (technical information are provided in the additional file n° 3).

## Results

Since 2013, 9.606 samples collected from wild animals (685 birds and 8.921 mammals) have been analyzed in the laboratories of the IZS-Teramo. More than 40% of samples have been collected from road killed animals. Two hundred seventy-four intentional animal poisoning have been recorded in the system, facilitating the reporting to the judicial authorities and the identification of highly risky areas to set up control and repression measures. Moreover, thanks to the monitoring health protocol, a canine distemper epidemic in wolves has been detected in 2013, in Abruzzo region, and monitored in its temporal and spatial evolution [16], as well as 4 cases of bovine tuberculosis in wild boars, between 2019 and 2022, in the two regions, which is a highly attentioned disease included in the eradication programme in domestic animals.

The system collects and returns the results of all the diagnostic tests performed on wild animals, moreover it allows tracking of suspected causes of death, including poisoning and road killing. Samples have been collected in all the region’s territories.

The user interface of the Web-GIS application designed to consult this data shows the basic map navigation tools, the administrative boundaries of Abruzzo and Molise regions overlaying on the default (topographic) basemap and the tools panel. The tools panel provides the users with two tabs: one for researching epidemiological data and the other for managing the layers on the map and some actions associated with them. The search tab shows a form with different fields and makes it possible to launch two different types of queries to the underlying database: health monitoring and cause of death. The fields of the search form are dynamically managed and adapted to the selected search type. The layers management tab provides the functions to turn the layers on and off, to open the associated legend, attribute table and statistics and to change the basemap.

When a search is performed, the results are shown at the same time as a set of points on the map, that can be clicked to display associated information, as an attribute table (downloadable as an Excel file for further investigations) and as a stacked histogram of their composition and distribution over time, to highlight all aspects of the information.

Each of the points returned represents a sample and can be shown in three different colors if the user has performed a health monitoring search:

- Red, for samples with a positive test result;
- Green, for samples with a negative test result;
- Grey, for other outcomes (inconclusive result, bad quality of sample collected).

If instead the user chooses to perform a search for cause of death, the points will be categorized as follows and the stacked histogram bars will reflect the same categories:

- Disease;
- Car accident;
- Poisoning;
- Edged weapon;
- Gun;
- Drowning;
- Other.

By clicking on the points it is possible to open a pop-up window that contains the main information of the sample, and allows downloading the result of diagnostic tests.

**Fig. 2:**
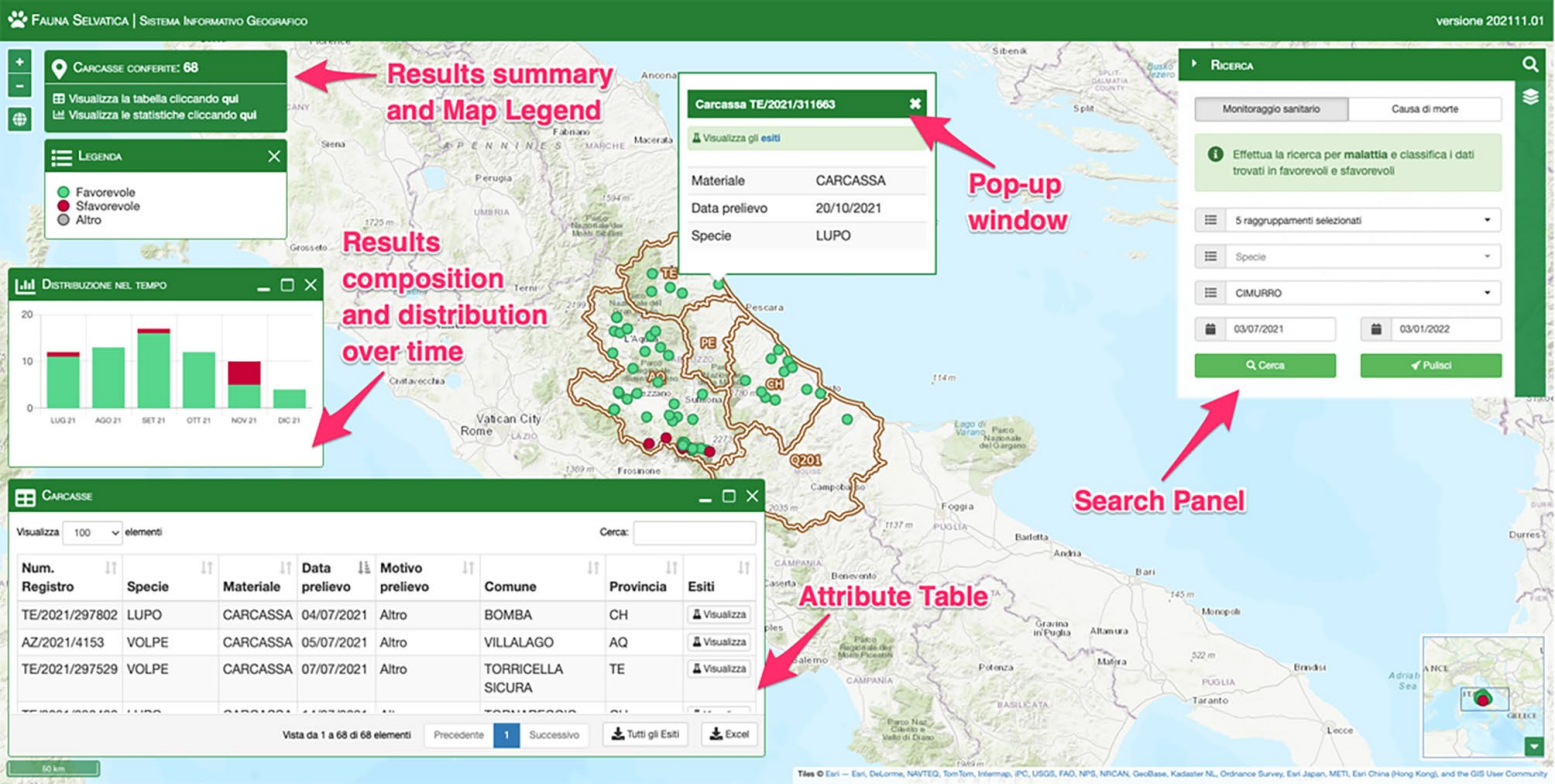
Health monitoring query results for distemper: location of the positive samples are marked as red points on the map, the attribute table provides additional information on the returned samples and gives the possibility to inspect the individual investigations performed on each of them. The histogram indicates the temporal distribution of samples, highlighting when positive and possibly time-concentrated results occurred. Two little widgets in the top-left corner display a results summary and the map legend. The Basemap throughout this figure were created using ArcGIS® software by Esri. ArcGIS® and ArcMap™ are the intellectual property of Esri and are used herein under license. Copyright © Esri. All rights reserved. For more information about Esri® software, please visit www.esri.com

## Discussion

One of the main difficulties in the study of the emergence of disease-causing infectious agents in wildlife is the lack of data about the prior absence of the agents (or a prior difference in disease dynamics) [4].

While it is more evident that the starting point, talking about knowing the health status of a wild population, is to choose the right sampling method, it is for sure less obvious that the information system in which this data is stored is equally important. In fact, not only the mere collection and storage of data, should be the aim of an information system, but also, and especially, it should give the possibility to consult it in an easy and instructive way.

We designed the Web-GIS monitoring Wildlife diseases for Abruzzo and Molise veterinary services, which are currently the only that can access to the application, to have a friendly and clean user interface, with a strictly selected set of tools designed to navigate and query the underlying spatial database [17]. This approach facilitates the data readability and accessibility and allows epidemiologists and decision makers to exploit the possibilities of GIS in a shared dimension using a web browser, without the need for specialized software on their computers and particular technical knowledge.

Thanks to the the Web-GIS monitoring Wildlife diseases for Abruzzo and Molise, the veterinary authorities were able to promptly identify cases of canine distemper, bovine tuberculosis, Newcastle disease, hepatitis E, Brucella suis, parvovirus, bluetongue, etc., in wild animals. After the detection of these diseases in wildlife, surveillance and control measures were implemented in the sympatric livestock in order to prevent spillover events. For example, a vaccination campaign against canine distemper was performed in livestock guardian dogs in critical areas where endangered species are kept, as well as clinical inspections in cattle farms in the surrounding areas where cases of tuberculosis in wild boar were confirmed. Therefore, the whole system developed proves to be a fundamental tool in an integrated surveillance for wildlife diseases.

In Italy there is a platform (Vetinfo portal) where several national information systems in the veterinary field are available and integrated to each other thanks to Web-Services. Within Vetinfo there are the National Database of Animals and Holdings (BDN), the National Information System for the Notification of Animal Diseases (SIMAN) [18], the national platform for the collection and analysis of complete genome sequences isolated from animal, food and environment (BioinfoDB) [13, 19].

SIMAN and the other information systems within the Vetinfo portal are able to produce reports and interactive thematic maps (mapping of infected holdings, listing of holdings in a buffer, etc.) thanks to the Web-GIS module. However, regarding wildlife disease data, only outbreak data/information from a national surveillance plan is currently available in SIMAN (i.e. West Nile disease, avian flu, rabies, African swine fever).

The integration of the Web-GIS described in this paper (intented as the data managed and the whole data workflow behind the scene) with the national information systems cited above and the complete acquisition of “denominator data” (size of the target populations), has begun in the year 2021/2022. It started with wild boar species, and in the near future, it will guarantee the development of an integrated and harmonized disease and population monitoring tool for wildlife. Moreover, thanks to the integration, it will be easier to explore, to study and promptly react in case of disease at the livestock-wildlife interface. Soon, access to other stakeholders will be given, such as national parks included in the area of interest and foresters, in order to optimize disease surveillance, road accident prevention, control and repression of crimes such as poisoning and poaching.

## Conclusion

Interactive maps allow immediately grasping the spatial relationships between the data itself and those between the data and the territory. They are particularly effective in supporting veterinary services to manage epidemic and non-epidemic emergencies and perform epidemiological investigations, but also to examine all control plans with a critical eye in order to identify new gaps and challenges. When interactive maps are accompanied by other visuals, such as interactive graphs capable of showing the non-geographical part of the information in an easily readable way, such as time trends, the result is a tool capable of showing in one go and in an extremely immediate way the main information relating to an ongoing phenomenon.

The system we described in this paper is used daily and efficiently by veterinary services in all those cases where multi-host diseases, such as bovine tuberculosis, may have a possible origin in domestic animals or may be a risk for them due to a spillover event.

It is worth noting that we have found that over time, since the veterinary services began to use the Web-GIS application, the quality of the geographic component of the data delivered to the Sample Acceptance Control Unit has gradually improved. When coordinates are missing or incorrect from a formal point of view, although there is no total loss of the data thanks to the automatic mechanism that replaces them with the centroid of the administrative unit, the generated approximation leads to a considerable loss of geographic information [20], making it almost useless in many cases. An application like the Web-GIS monitoring Wildlife diseases for Abruzzo and Molise makes the benefits of precise geographic information tangible and helps the users to understand that the higher the quality and completeness of the data supplied, the more the system is able to provide precise and useful answers for the analysis of current phenomena.

## Electronic supplementary material

Below is the link to the electronic supplementary material.

Supplementary Material 1: Laboratory Requisition Form

Supplementary Material 2: Health monitoring protocol

Supplementary Material 3: System architecture and software components

## Data Availability

Not applicable.

## Abbreviations

GIS: Geographic Information System
DBMS: Database Management System
WAHIS: World Animal Health Information System
WOAH: World Organisation for Animal Health
LRF: Laboratory Requisition Form
SILAB: Laboratory Information Management System of the IZS-Teramo
ISTAT: (Italian) National Institute of Statistics
AU: Administrative Unit
ReST: Representational State Transfer
J2EE: Java 2 Enterprise Edition
BDN: National Database of Animals and Holdings
SIMAN: National Information System for the Notification of Animal Diseases
