# Supplementary material for: A web-based geographic information system monitoring wildlife diseases in Abruzzo and Molise regions, Southern Italy
Source: BMC Vet Res. 2023 Oct 2;19:183. doi: 10.1186/s12917-023-03727-9 (PMC10544395; doi:10.1186/s12917-023-03727-9)
Supplement: Supplementary file 1 — Supplementary Material 1: Laboratory Requisition Form [file 12917_2023_3727_MOESM1_ESM.pdf]

UNDER SEIZURE ❑ Yes ❑ No

DATE ___/___/_____

WILDLIFE INFORMATION SYSTEM

LABORATORY REQUISITION FORM

ANIMAL IDENTIFICATION

SPECIES	IDENTIFIER	SEX	AGE	MATERIAL
	1.	❑ M<br>❑ F<br>❑ NA		❑ Carcass ❑ Muscle ❑ Lung ❑ Heart ❑ Spleen<br>❑ Kidney ❑ Liver ❑ Intestine ❑ Feces<br>❑ Gastric contents<br>❑ Blood ❑ Serum<br>❑ Lymph nodes ____________________<br>Other __________________________
	2.	❑ M<br>❑ F<br>❑ NA		❑ Carcass ❑ Muscle ❑ Lung ❑ Heart ❑ Spleen<br>❑ Kidney ❑ Liver ❑ Intestine ❑ Feces<br>❑ Gastric contents<br>❑ Blood ❑ Serum<br>❑ Lymph nodes ____________________<br>Other __________________________
	3.	❑ M<br>❑ F<br>❑ NA		❑ Carcass ❑ Muscle ❑ Lung ❑ Heart ❑ Spleen<br>❑ Kidney ❑ Liver ❑ Intestine ❑ Feces<br>❑ Gastric contents<br>❑ Blood ❑ Serum<br>❑ Lymph nodes ____________________<br>Other __________________________
	4.	❑ M<br>❑ F<br>❑ NA		❑ Carcass ❑ Muscle ❑ Lung ❑ Heart ❑ Spleen<br>❑ Kidney ❑ Liver ❑ Intestine ❑ Feces<br>❑ Gastric contents<br>❑ Blood ❑ Serum<br>❑ Lymph nodes ____________________<br>Other __________________________

COLLECTION PLACE

❑ Natural habitat ❑ Conservation facility ❑ Rescue centre

❑ Other ❑ Holding

Holding Identifier □□□ □□ □□□

Province ________________________________

City________________________________

Toponym ________________________________

Geographic coordinates in decimal degrees(WGS84)

LATITUDE □□.□□□□□

LONGITUDE □□.□□□□□

CLIENT

Institution ________________________________

Address ________________________________

Responsible Vet________________________________

____

Telephon ________________________________

E-mail ________________________________

ANAMNESIS

____

____

SAMPLING CONTEST

❑ INVESTIGATION ON THE CAUSE OF DEATH

❑ HUNTING ❑ SELECTIVE HUNTING / CAPTURE CAGES

❑ CAPTURE / KILLING FOR STUDY PURPOSE ❑ MOVING LIVE ANIMALS

❑ LIVE ANIMAL WITH SYMPTOMS

❑ OTHER______________________________________

SUSPECTED CAUSE OF DEATH

❑ POISONING ❑ DROWNING ❑ ROAD KILLED ❑ GUNSHOT WOUNDS ❑ DISEASE

❑ OTHER _______________________

CLINICAL SYMPTOMS

❑ SKIN ❑ SENSE ORGANS ❑ RESPIRATORY SYSTEM ❑ GASTROINTESTINAL APPARATUS

❑ NERVOUS SYSTEM ❑ REPRODUCTIVE SYSTEM ❑ MUSCULOSKELETAL SYSTEM

❑ CARDIOVASCULAR SYSTEM ❑ LYMPHATIC SYSTEM

TEST REQUIRED

❑ NECROPSY ❑ COMPLETE BLOOD COUNT ❑ CLINICAL BIOCHEMISTRY

❑ SEROLOGICAL ______________________________

❑ TOXICOLOGICAL ______________________________

❑ BACTERIOLOGICAL ______________________________

❑ PARASITOLOGICAL______________________________

❑ VIROLOGICAL ______________________________

❑ HISTOPATHOLOGY ______________________________

CARCASS REQUEST

Do you intend to request the return of the carcass?

❑ Yes ❑ No

❑ whole carcass ❑ Head ❑ Head and Skin

❑ Other______________________

Date____/____/__________ **Signature**______________________________
