# Supplementary material for: A web-based geographic information system monitoring wildlife diseases in Abruzzo and Molise regions, Southern Italy
Source: BMC Vet Res. 2023 Oct 2;19:183. doi: 10.1186/s12917-023-03727-9 (PMC10544395; doi:10.1186/s12917-023-03727-9)
Supplement: Supplementary file 3 — Supplementary Material 3: System architecture and software components [file 12917_2023_3727_MOESM3_ESM.docx]

System Architecture and Software components

The Web-GIS application described in this paper is based on both open source and proprietary solutions and relies on a typical client-server architecture represented in the figure below.


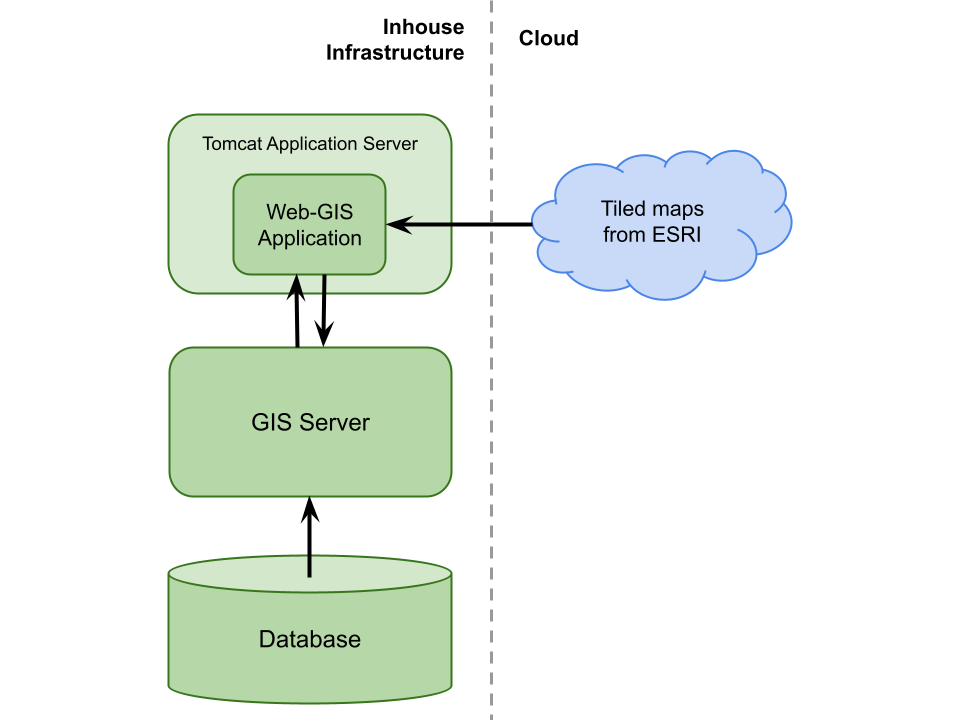


# Data storage and Back-end components

Data is stored in-house in an Oracle Relational DataBase Management System (RDBMS) (Oracle Database 12c Enterprise Edition Release 12.2.0.1.0 - 64bit Production). Wildlife data are stored in a dedicated db schema, connected with an internal schema of the Laboratory Information Management System of the IZS-Teramo (SILAB), collecting all data of accepted samples and their life cycle up to their final outcomes and test reports.

A nightly batch procedure read the original data on SILAB and updates two views on the Wildlife database: one containing data of sampling (with also additional data characterizing the sample or sampled animal and useful for epidemiological analysis, such as “free life/animal yes-no, finding circumstances, reason of death, presence of photo yes-no, place of finding, clinical signs presence, sampling reason, animal gender and age”), and a second view containig laboratory tests outcomes.

The Wildlife database is connected to an Esri ArcGIS Server 10.8.1, that is in charge of sharing a Representational State Transfer (ReST) web service, secured using the built-in token based authentication functionality.

More technical details and specifications about the back-end stack can be found at the following addresses:

- Oracle, <https://www.oracle.com/database/>
- ArcGIS Server, <https://enterprise.arcgis.com/en/server/10.8/get-started/windows/what-is-arcgis-for-server-.htm>
- ArcGIS tokens, https://enterprise.arcgis.com/en/server/10.8/administer/windows/about-arcgis-tokens.htm

# Front-end components

We built from scratch a JavaScript front-end based on the OpenLayers, jsPanel, DataTables and Chart.js open source libraries and styled using the open source Bootstrap CSS Framework.

This application shows the geographic and epidemiological data coming from the in-house back-end (DBMS and ReST) and allows the users to fully interact with it, while for the basemaps it relies on the cloud service offered by Esri Inc., which provides freely accessible global topographic, road, gray canvas and satellite tiled maps.

The Web-GIS application is finally deployed on an Apache Tomcat Application Server.

More in detail:

- OpenLayers was used to render the map and the spatial data, and to build the interactions with the spatial objects. Docs at: <https://openlayers.org/doc/>.
- jsPanel was used to create the floating windows showing the search panel, the tables and the charts. Library docs at: <https://jspanel.de/#documentation>.
- DataTables was used to render the data coming from the server as interactive tables. Docs at: <https://datatables.net/manual/>.
- Chart.js was used to render the data coming from the server as interactive charts. Docs at: <https://www.chartjs.org/docs/latest/>.
- Bootstrap was used to harmonize and give graphical consistency to the whole application. Docs at: <https://getbootstrap.com/docs/5.3/getting-started/introduction/>.
- Apache Tomcat Application Server was used to deploy the application, packed in .war format. Docs at: <https://tomcat.apache.org/>

Esri basemaps used in the Web-GIS application are listed below, using the form recommended in the official Esri FAQ for citations (<https://support.esri.com/en-us/knowledge-base/faq-what-is-the-correct-way-to-cite-an-arcgis-online-ba-000012040>):

Esri. "Topographic" [basemap]. Scale Not Given. "World Topographic Map". August 3, 2021. <http://www.arcgis.com/home/item.html?id=30e5fe3149c34df1ba922e6f5bbf808f> (July 13, 2023).

Esri. “Road” [basemap]. Scale Not Given. “World Street Map”. August 3, 2021. <https://www.arcgis.com/home/item.html?id=3b93337983e9436f8db950e38a8629af> (July 13, 2023)

Esri. “Gray” [basemap]. Scale Not Given. “World Light Gray Base”. July 1, 2021. <https://www.arcgis.com/home/item.html?id=ed712cb1db3e4bae9e85329040fb9a49> (July 13, 2023)

Esri. “Satellite” [basemap]. Scale Not Given. “World Imagery”. July 5, 2023. <https://www.arcgis.com/home/item.html?id=10df2279f9684e4a9f6a7f08febac2a9> (July 13, 2023)
